# Average genome size estimation improves comparative metagenomics and sheds light on the functional ecology of the human microbiome

**DOI:** 10.1186/s13059-015-0611-7

**Published:** 2015-03-25

**Authors:** Stephen Nayfach, Katherine S Pollard

**Affiliations:** Integrative Program in Quantitative Biology, University of California, San Francisco, CA 94158 USA; Gladstone Institutes, San Francisco, CA 94158 USA; Institute for Human Genetics, University of California, San Francisco, CA 94158 USA; Department of Epidemiology and Biostatistics, University of California, San Francisco, CA 94158 USA

## Abstract

Average genome size is an important, yet often overlooked, property of microbial communities. We developed MicrobeCensus to rapidly and accurately estimate average genome size from shotgun metagenomic data and applied our tool to 1,352 human microbiome samples. We found that average genome size differs significantly within and between body sites and tracks with major functional and taxonomic differences. In the gut, average genome size is positively correlated with the abundance of *Bacteroides* and genes related to carbohydrate metabolism. Importantly, we found that average genome size variation can bias comparative analyses, and that normalization improves detection of differentially abundant genes.

**Electronic supplementary material:**

The online version of this article (doi:10.1186/s13059-015-0611-7) contains supplementary material, which is available to authorized users.

## Background

Shotgun metagenomics is increasingly being used to characterize the functional composition of microbial communities from the human body and many environments [1-4]. A common goal of these studies is to quantify gene family abundance and identify microbial genes or pathways that differ in abundance between environments, host phenotypes, or experimental conditions. Comparative metagenomics has shed light on how microbes have functionally adapted to the myriad of environments on Earth [4], and how variation in the functional composition of microbial communities can impact human health and disease [5,6]. Functional variation can be interpreted in the context of taxonomic variation estimated from the same shotgun data, shedding light on evolutionary and ecological processes. These studies require accurate quantification of gene abundance from microbial community DNA.

While often overlooked, it is important to consider the average genome size (AGS) of cells in a microbial community when performing comparative metagenomic analyses. From a statistical perspective, AGS can be a potential source of bias when comparing the abundance of genes between communities. Specifically, the probability of sampling a gene from a community is inversely proportional to the AGS of that community [7]. If not accounted for, differences in AGS between samples can lead to the appearance of variation among genes that are present at equal copy number per cell (that is, false positives) and the appearance of stability among genes that vary in copy number per cell (that is, false negatives). Along with library size (that is, sequencing depth) and gene length, AGS should be accounted for in comparative metagenomic studies to identify ecologically meaningful genomic differences between microbial communities.

AGS is also important for understanding the ecological and evolutionary forces acting on microorganisms within an environment. From an ecological perspective, microbial genome size may reflect environmental complexity, metabolic lifestyle, and community niche [8-12]. For example, in the gut it is believed that organisms with larger genomes follow more generalist lifestyles whereas those with smaller genomes are more specialized [8]. To illustrate this, *Bacteroides thetaiotamicron* (6.5 Mb) has a large genome with the metabolic potential to utilize a variety of both diet and host-derived glycans, whereas *Methanobrevibacter smithii* (1.9 Mb) is a methanogen specialized for utilization of H_2_. From an evolutionary perspective, reduced genome size may reflect genetic drift in small populations or genome streamlining in large populations [13,14]. Genetic drift can shape the size of microbial genomes by the fixation of mutations, which are biased towards deletions in Bacteria [15]. In contrast, genome streamlining can promote reduced genome size by selecting for the efficient use of nutrients in large microbial populations where nutrients limit growth [14].

Up to this point it has not been possible to rapidly and accurately estimate the AGS of microbial communities due to a lack of software designed for modern metagenomics data. This has limited our understanding of both the extent and impact of AGS variation in many environments, including the human microbiome. The only publicly available software tool, GAAS [10], estimates AGS based on BLAST searches of shotgun sequences against a database of microbial genomes. Given the large and increasing scale of both metagenomic data and reference genomes, and the relatively slow speed of BLAST, this method is not computationally practical (Results). Additionally, microbial communities frequently contain high fractions of 'novel' organisms, which have not been cultured or sequenced. Even in the well-studied human gut microbiome, it has been estimated that, on average, 43% of species abundance and 58% of richness cannot be captured by current microbial reference genomes [16]. It is not clear whether GAAS is able to accurately estimate AGS for metagenomes composed of novel taxa. Raes *et al*. [9] proposed to address these issues by estimating average genome size based on the density of reads assigned to a set of 35 essential single-copy genes using BLASTX. While significantly faster and less dependent upon community composition, this method was not designed for reads shorter than 300 base pairs (bp) and no software was released. Although current sequencing technologies are beginning to generate longer reads, there remains a huge volume of existing short-read data; for example, the MetaHIT [1,17-19] and Human Microbiome Project (HMP) [20] projects have together generated over 130 billion metagenomic reads from the human microbiome with an average length of only 96 bp [21]. It is not clear whether the Raes method is able to estimate AGS for these modern short-read libraries. Others [22,23] have described similar methods, but these have not been extensively validated or made available as software.

To address this problem, we developed MicrobeCensus to rapidly and accurately estimate AGS from shotgun metagenomic data and applied our tool to 1,352 human microbiome samples. We adopted a similar approach to Raes *et al*., but made significant methodological improvements that allowed us to accurately estimate AGS using reads as short as 50 bp. We found that AGS differs significantly both within and between body sites in the human microbiome and tracks with major functional and taxonomic differences between communities. For example, the AGS of stool metagenomes ranged from 2.5 to 5.8 megabases (Mb) and was positively correlated with the abundance of *Bacteroides* and genes related to metabolism, biosynthesis, and two-component systems, whereas Firmicutes and genes related to membrane transport were more abundant in metagenomes with smaller AGS. Furthermore, we confirmed that AGS is a major bias in comparative metagenomic analyses, and that normalization improves detection of differentially abundant genes. These discoveries could not have been made without the development of MicrobeCensus.

## Results and discussion

### Estimation of average genome size from metagenomic data

At the core of our method is a database of 30 essential single-copy gene families found in nearly all Bacteria and Archaea (Additional file 1). These are a subset of the 40 bacterial and archaeal PhyEco markers identified by Wu, Jospin and Eisen [24], and they largely overlap with gene sets previously identified by Ciccarelli *et al.* [25], Raes *et al.* [9], and Wu *et al.* [26]. A preliminary analysis shows that most of these gene families are also present in Fungi, although are not as universal or stable as in Bacteria and Archaea (Additional files 1 and 2). Hence, we can expect that the vast majority of cells in a microbial community - particularly those dominated by Bacteria or Archaea - will carry one copy of each of these genes.

Because of this unique property, these 30 genes can be used to estimate the average genome size of cells in a microbial community based on metagenomic sequencing data. This is the approach used by Raes and others [9,22,23]. Specifically, the AGS of a community will be inversely proportional to the relative abundance, *R*, of an essential single-copy gene in that community: *AGS* ∝ *R*^-1^. In other words, these essential genes will be sequenced at a higher rate in a community with a small AGS relative to a community with a large AGS; this is simply because these genes make up a larger fraction of the total genomic DNA in the community with smaller genomes.

To implement this approach, we had to solve three problems. First, we had to optimize parameters for mapping metagenomic reads to the database of essential genes in order to minimize the technical variation of *R* between metagenomes (Additional file 3). The mapping occurs via sequence homology search, using RAPsearch2 [27], a fast alternative to BLAST. We found that the best mapping parameters depended on read length and, to a lesser extent, the identity of the gene family, which is a problem that has been previously described for taxonomic classification of metagenomic reads [28]. Second, we needed to estimate the proportionality constant, *C*, between *AGS* and *R*^-1^. This constant cannot be determined *a priori* and depends upon read length, mapping parameters, and the gene family (Additional file 3). Third, gene families vary in their usefulness for estimating AGS, due to occasional deviations from having exactly one copy per cell, as well as variability in the accuracy with which reads are mapped to each family. Hence, when averaging AGS estimates across the 30 gene families, we sought to develop a method to weight each family so that those with high accuracy contributed more to the combined AGS estimate than did those with low accuracy. To address each of these issues, we conducted a large series of shotgun sequencing simulations in which the AGS of each library was known. This enabled us to optimize mapping parameters, identify proportionality constants, and estimate gene-family weights (Materials and methods).

**Figure 1:**
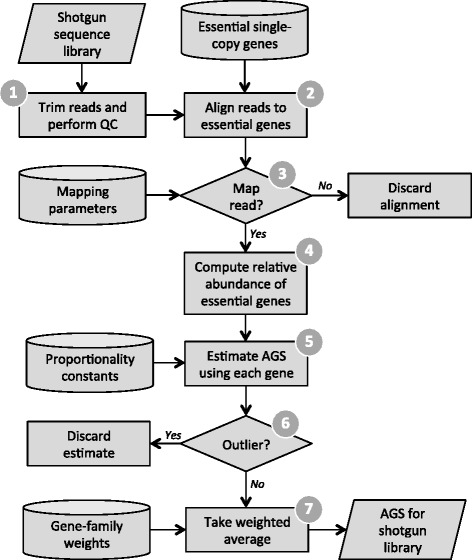
**Flowchart for estimating AGS from a shotgun metagenome.** 1) MicrobeCensus takes the first *n* reads of at least *i* base pairs from the shotgun metagenome and trims these reads down to *i* base pairs. 2) These reads are aligned against the database of essential genes using RAPsearch2. 3) A read is mapped to an essential gene family, *j*, if its top scoring alignment satisfies the mapping parameters, which are optimized for gene *j* and read length *i*. 4) Based on these mapped reads, the relative abundance of each essential gene family, *R*_*j*,_ is computed. 5) Next, we use *R*_*j*_ to obtain an estimate of AGS for each gene. 6) Outlier predictions are removed and 7) MicrobeCensus takes a weighted average over the remaining estimates to produce a robust estimate of AGS for the shotgun metagenome. QC, quality control.

The resulting new method, called MicrobeCensus, rapidly and accurately estimates AGS from metagenomic data (Figure 1). MicrobeCensus first downsamples the first *n* reads of at least *i* base pairs from the metagenome, which we found improves computational efficiency without sacrificing accuracy (Additional file 4; Results). Next, these reads are trimmed from their 3′ end down to *i* bp. This is principally done because our method uses parameters that are read-length specific. Next, these reads are translated and aligned against the database of essential genes using RAPsearch2. Reads are classified into a gene family if their top scoring alignment meets or exceeds the optimal mapping parameters for that gene family and the specified read length. We then obtain an estimate of AGS for each gene family based on that family’s relative abundance and proportionality constant. Finally, MicrobeCensus eliminates any outliers and take a weighted average over the remaining estimates to produce a robust estimate of AGS for the metagenome. We found that the 30 gene families we selected were sufficient to produce accurate estimates of AGS, and additional genes would probably not have significantly improved performance (Additional file 5; Materials and methods).

MicrobeCensus is provided as a command line software package written in Python. Software, examples and documentation are freely available at [29]. Additionally, we have provided software necessary to retrain MicrobeCensus using user-supplied gene families and training genomes. This may be important as new genomic data become available or for researchers who wish to train MicrobeCensus for microbes from specific environments or using additional gene families; however, retraining will not be necessary for most applications.

### Comparison of MicrobeCensus with existing methods

Because MicrobeCensus estimates average genome size using a set of genes present in nearly all Bacteria, Archaea, and Fungi, we hypothesized that our tool would be robust to high proportions of novel taxa present in metagenomes. Conversely, we suspected that methods that rely on reference genomes to estimate AGS would not perform as well in these cases. To this end, we benchmarked MicrobeCensus against the tool GAAS [10] on 20 simulated metagenomes composed of 100-bp reads from prokaryotic microbial communities (Additional file 6; Materials and methods). To simulate the presence of novel taxa, we held-back reference sequences belonging to organisms from the same taxonomic group as organisms in the metagenome, and estimated AGS for each method using the remaining reference sequences. For MicrobeCensus, the reference sequences included the 30 gene families, whereas the reference sequences for GAAS were complete microbial genomes. We performed this procedure and evaluated performance at each taxonomic level: species, genus, family, order, class, and phylum. For example, at the genus level, this procedure would discard alignments between *Escherichia coli* shotgun sequences and all reference sequences from *Escherichia*; at the phylum level, this procedure would discard alignments between *E. coli* shotgun sequences and all reference sequences from Proteobacteria.

**Figure 2:**
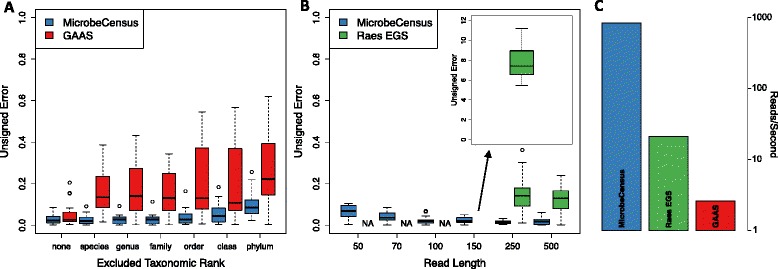
**Comparison of MicrobeCensus to existing methods. (A,B)** Performance of MicrobeCensus was compared with that of existing methods using 20 simulated metagenomes. Unsigned error is defined as: |*AĜS* - *AGS*|/*AGS*. (A) MicrobeCensus versus GAAS at different levels of taxonomic exclusion. To simulate the presence of novel taxa, we held back reference sequences belonging to organisms from the same taxonomic group as organisms in the metagenome, which is indicated on the x-axis. 'None' indicates that no reference sequences were held back. Metagenomes were composed of 100-bp reads. (B) Estimation error for MicrobeCensus versus the method described by Raes *et al*. [9] for metagenomes of various read length. 'NA' indicates that AGS could not be estimated. **(C)** Speed (reads/second) of MicrobeCensus compared with existing methods on a simulated 150-bp library.

We quantified AGS estimation accuracy using the median unsigned error, which summarizes absolute errors to account for both over- and under-estimation. When we did not exclude any reference sequences, both GAAS and MicrobeCensus performed well for the 20 datasets (labeled 'none' in Figure 2A), indicating that both methods can accurately estimate AGS for metagenomes composed of taxa that are represented in the reference database. However, when species from the metagenome were excluded from the reference database, the median unsigned error for GAAS increased to 13.5%, while error for MicrobeCensus remained 2% and did not rise above 3% even at higher levels of taxonomic exclusion, confirming our hypothesis that MicrobeCensus would be robust to the presence of novel taxa. Using MicrobeCensus, we were able to obtain reasonable estimates of AGS even for metagenomes composed of taxa with no representatives in the reference database at the phylum level (8.6% median unsigned error), while error for GAAS was over 20%.

We also compared the performance of MicrobeCensus against the method described by Raes *et al*. [9], who were the first to use essential single copy genes to estimate average genome size from metagenomic data. However, this method only considered one set of parameters for determining whether a read mapped to an essential gene - regardless of the read length or the target gene family - and did not evaluate performance on read lengths shorter than 300 bp. Because we found that mapping parameters needed to be tuned depending on read length, we hypothesized that this method would not be able to accurately estimate AGS for modern short-read libraries. To evaluate this, we implemented the method described by Raes *et al*. and benchmarked this method against MicrobeCensus using simulated metagenomes composed of reads ranging from 50 to 500 bp (Materials and methods). We found that the Raes method was unable to generate any estimates of AGS for the short-read libraries (≤100 bp) due to a complete lack of alignments that satisfied the method’s mapping parameters (labeled as 'NA' in Figure 2B). In other words, the mapping parameters were too strict for the short-read libraries. For intermediate read lengths (150 bp), the method was able to generate estimates of AGS, but was not accurate (all > 500% unsigned error) and overestimated AGS. As expected, long-read libraries (250 to 500 bp) produced moderately accurate estimates (13 to 14% median unsigned error), although tended to underestimate AGS. In contrast, MicrobeCensus was able to accurately estimate AGS across all libraries, using reads as short as 50 bp (all < 7% median unsigned error), but particularly for reads at least 100 bp long (all ≤2% median unsigned error).

### The effect of sequencing error on estimation accuracy

**Figure 3:**
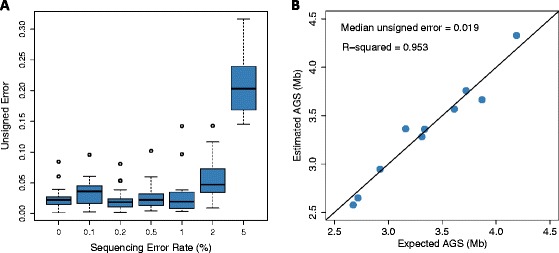
**The effect of sequencing error on estimation accuracy.** Unsigned error is defined as: |*AĜS* - *AGS*|/*AGS*. **(A)** MicrobeCensus was used to estimate the AGS of 20 metagenomes that were simulated with up to a 5% sequencing error rate. Metagenomes were composed of 100-bp reads from prokaryotes. **(B)** MicrobeCensus was used to estimate the AGS of 10 metagenomes that were composed of real Illumina reads pooled from 10 randomly chosen isolate sequencing projects.

While we were able to obtain accurate estimates of average genome size for error-free libraries, it was not clear if we could accurately estimate AGS from libraries that contained sequencing error, artificially duplicated reads, and a non-uniform distribution of coverage. To evaluate the effect of sequencing error, we simulated 100-bp metagenomes with up to 5% sequencing error rates (Materials and methods). We found that we could accurately estimate AGS from libraries that contained up to 2% sequencing error - beyond this, estimation error quickly increased (Figure 3A). Luckily, most current sequencing platforms have raw error rates below 2%, including Illumina MiSeq (0.80%), Ion Torrent PGM (1.71%), Illumina GAIIx (0.76%), Illumina HiSeq 2000 (0.26%), and 454 GS-FLX Titanium (1.07%) [30,31].

Next, we built metagenomes composed of real Illumina reads from completed microbial isolate sequencing projects (Additional file 7; Materials and methods) and used MicrobeCensus to estimate AGS for each of these mock metagenomes. Overall, we estimated AGS with 1.9% median unsigned error and less than 0.1% median signed error, indicating that we are able to obtain accurate, unbiased estimates of AGS from real Illumina libraries (Figure 3B). However, our estimates of the size of each of the 42 individual genomes were less accurate (6.4% median unsigned error when applying MicrobeCensus to single sequencing projects; Additional file 7), suggesting that it is actually easier to estimate AGS in more complex communities. We confirmed this hypothesis with simulations (Additional file 8).

Finally, we were interested in exploring how various quality control procedures could improve estimation accuracy in real datasets (Materials and methods). Interestingly, we found that while removing adaptor contamination and filtering duplicate reads resulted in a marginal reduction in estimation error, quality-filtering reads had very little benefit and in some cases actually reduced accuracy (Additional file 9), which may be due to a biased distribution of quality scores [32]. Regardless, MicrobeCensus includes options for filtering duplicate and low quality reads.

### Estimation accuracy in the presence of microbial eukaryotes and viruses

**Figure 4:**
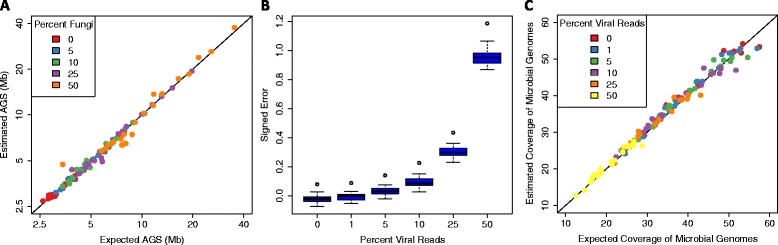
**Estimation accuracy in the presence of microbial eukaryotes and viruses. (A)** MicrobeCensus was used to estimate the AGS of 20 simulated 100-bp metagenomes that contained up to 50% of Fungi, representing up to 94% of total reads. Note that axes are plotted on a log scale. **(B)** MicrobeCensus was used to estimate the AGS of 20 simulated 100-bp metagenomes that contained up to 50% of reads from viruses. Signed error is defined as: (*AĜS* - *AGS*)/*AGS*. **(C)** AGS estimates from (B) were used to estimate the total coverage of microbial genomes present in the simulated metagenomes. Estimated coverage of microbial genomes was obtained by dividing the number of total base pairs in a metagenome by the estimated AGS for that metagenome.

Next, we evaluated whether accurate estimates of AGS could be made in the presence of microbial eukaryotes. Fungi are typically minority members of human microbiome communities but can occasionally constitute a significant proportion of metagenomic libraries [1,20,33]. To address this, we simulated 20 communities in which 0 to 50% of genomes were Fungi and used MicrobeCensus to estimate AGS for these communities (Additional file 6; Materials and methods). Fungal genome sizes ranged from 2.5 to 66.3 Mb with an average of 20.4 Mb. We used Fungi as a proxy for microbial eukaryotes due to the availability of complete genome sequences and the presence of these taxa in the human microbiome [34,35]. Because microbial eukaryotes were not included in our database and were not used to train MicrobeCensus, we wondered if the presence of these taxa would lead to inaccurate estimates of AGS. Surprisingly, AGS estimates for most of these communities were quite accurate, although not as accurate as for the bacterial and archaeal communities (Figure 4A). Even when Fungi were at 50% relative abundance, representing, on average, 79% of total reads, the median unsigned error was only 5% for the 20 communities. Nonetheless, in the future, when more complete genome sequences of microbial eukaryotes are available, particularly for Protists, it should be possible to retrain MicrobeCensus to achieve optimal performance for these types of communities. We have included training code in our software package for such extensions.

Although MicrobeCensus achieved good performance for the fungal communities, it was not clear what affect DNA viruses would have. While viruses outnumber microbial cells 10:1 in most environments, it is believed that viral DNA represents only 2 to 5% of the total DNA in microbial communities [36]. However, this may be an underestimate owing to a lack of homology to reference databases (for example, NCBI nr). For example, a recently discovered bacteriophage from human stool was found to represent up to 22% of the total DNA [37]. To quantify the effect of viral reads on AGS estimation, we simulated 20 metagenomes that were composed of between 0 and 50% of reads from phage genomes and used MicrobeCensus to estimate AGS for these communities (Additional file 6; Materials and methods). Note that the expected AGS for these communities is based on the genome sizes of cellular microbes and does not account for the genome sizes of viruses. We found that moderate levels of viral reads were tolerated, but that very high levels caused MicrobeCensus to overestimate AGS for the communities (Figure 4B). For example, estimation error was ≤3% for communities containing ≤5% viral reads, but increased to approximately 9% when viral reads comprised 10% of the metagenome. Because viral reads are not homologous to the 30 essential gene-families used by MicrobeCensus, their presence decreases the fraction of reads mapped to these genes and creates the impression of a larger AGS. Therefore, MicrobeCensus may not be appropriate for estimating the AGS of communities that *a priori* are known to contain high proportions of viral reads (for example, purified virus-like particles or small size fractions from seawater). Researchers concerned with this issue could screen and remove viral sequences from their metagenome prior to AGS estimation.

While AGS was overestimated in the presence of viruses, we were still able to accurately estimate the total coverage of cellular microbes present in the simulated metagenomes (Figure 4C). This value is obtained by dividing the total number of sequenced base pairs by the estimated AGS of the metagenome. This value represents the number of microbial genome equivalents present in a metagenome and can be used to normalize the abundance of gene families across metagenomics experiments (Results and Materials and methods). While viruses may contribute to the metabolic potential of microbial communities, their functions are generally not captured by current protein family databases used for inferring community function (for example, KEGG Orthology [38], eggNOG [39], FIGfams [40]), which only include orthologs from Bacteria, Archaea, and Eukaryota. Hence, viral reads do not typically contribute to the total count of reads for a protein family, and therefore AGS estimated by MicrobeCensus can still be used to normalize protein abundance profiles.

### Software speed

Due to the increasingly large scale of metagenomics data, it is important to be able to process datasets in a reasonable time frame, particularly for users with limited computing resources. Therefore, we benchmarked the speed of MicrobeCensus and other methods on a simulated 150-bp shotgun sequence library (Figure 2C). We found that MicrobeCensus was approximately 320 times faster than GAAS and approximately 40 times faster than our implementation of the Raes method. When using a single CPU, MicrobeCensus was able to process about 830 reads/second, or about 1 million reads in 20 minutes. Conversely, it took GAAS 10.2 hours to process only 100,000 reads. Using multi-threading we were able to further increase the speed of MicrobeCensus: 1.5 times for 2 cores, 2.0 times for 4 cores, and 2.5 times for 8 cores. Most of the difference in speed between these methods is a result of the fact that MicrobeCensus searches reads against a database of only 30 genes, which together comprise less than 1% of most bacterial genomes, and the difference in speed in the underlying sequence alignment algorithms - MicrobeCensus utilizes RAPsearch2, which is approximately 20 to 90 times faster than BLAST [27].

Additionally, we were interested in determining the fewest number of reads MicrobeCensus needed for a precise estimate of AGS. We reasoned that we might be able to precisely estimate AGS using only a small fraction of large datasets, which would reduce the runtime of MicrobeCensus. On the other hand, certain datasets might not be large enough to obtain a reliable estimate of AGS. To address this question, we ran MicrobeCensus on several samples from the HMP [20], using between 10,000 and 20 million randomly sampled reads (Materials and methods). For each sample, this allowed us to estimate the amount of dispersion (that is, variability in AGS estimates) at a given number of sampled reads. For all HMP samples, we found that dispersion was sufficiently low at 300,000 reads and reached an asymptote at about 500,000 reads (Additional file 4). At sampling depths below 300,000 reads, dispersion quickly increased. The HMP samples we selected had estimated AGS values that ranged from 1.8 to 4.3 Mb - it will likely be necessary to use more than 300,000 reads to achieve the same level of dispersion for metagenomes with larger AGS. In summary, MicrobeCensus is able to generate low variance estimates of AGS from typical metagenomic datasets using as few as 300,000 to 500,000 reads with a runtime of approximately 10 minutes on a single CPU.

### Average genome size varies systematically in human microbiome data

To survey natural variation of average genome size in the human microbiome, we used MicrobeCensus to estimate the AGS of 1,352 metagenomic samples from human subjects, collectively spanning five major body sites and five countries (Additional file 10). Included in this analysis were samples from the HMP and three other large studies of the human gut microbiome [1,5,6].

**Figure 5:**
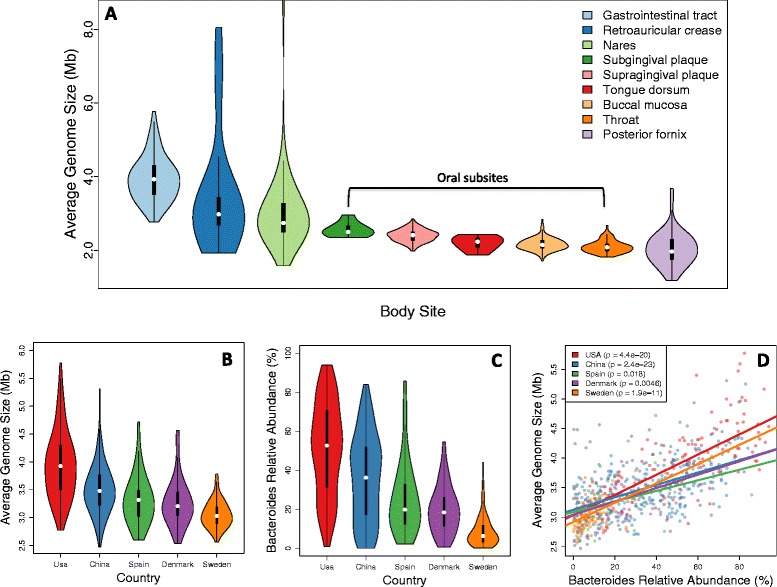
**Average genome size varies systematically in human microbiome data. (A)** Distribution of estimated AGS for 736 samples from the Human Microbiome Project. **(B)** Distribution of estimated AGS for 725 stool samples obtained from subjects originating from five different countries. **(C)** Estimated relative abundance of *Bacteroides* for the same samples shown in (B). **(D)** Country-specific correlations between AGS and *Bacteroides* relative abundance for stool samples.

We found significant differences in AGS between nearly all body sites within the HMP dataset (Figure 5A; Additional file 11). The stool communities had an especially large and broad range of AGS (2.8 to 5.8 Mb, mean = 3.9 Mb), which may reflect adaptation to variability in the human diet and rapid changes in the availability of nutrients. This is also consistent with previous reports that stool communities have the highest ratio of genes per operational taxonomic unit [20], and that several prominent members of the gut have large genomes, including *B. thetaiotamicron* (6.5 Mb), *B. ovatus* (6.5 Mb), and *B. vulgatus* (4.9 Mb). The skin and nares communities had intermediate AGS, but each contained several outliers greater than 6 Mb, while the oral and urogenital communities were characterized by the lowest AGS (mean = 2.23 and 2.11 Mb, respectively). For example, only 3 of the 396 oral samples had an estimated AGS that exceeded any of the 146 stool samples. Furthermore, we found that AGS was remarkably stable within each of the oral sites - the maximum coefficient of variation in any of the oral sites was only 0.08, in contrast to high values in stool (0.16), urogenital tract (0.23), airways (0.36), and skin (0.45) - which may be due to a combination of factors, including lower beta diversity, greater functional convergence, and less environmental variability, although these possibilities need to be investigated in greater detail.

We found that differences in AGS within and between body sites could be largely explained by genus and species level taxonomic variation of Bacteria (Additional file 12). Using a multiple linear model of species-level relative abundance, we were able to explain 74% of the total variation in AGS across all HMP samples. Within the HMP stool communities, we found that AGS was positively correlated with abundant *Bacteroides* spp., including *Bacteroides ovatus* (6.5 Mb), *B. thetaiotaomicron* (6.3 Mb), and *Bacteroides xylanisolvens* (6.0 Mb) (all *P* < 1e-4, all r^2^ > 0.12). In contrast, members of the order Clostridiales (*P* = 1.5e-7, r^2^ = 0.18) were negatively correlated with AGS, consistent with reports that Firmicutes possess smaller genomes and a disproportionately smaller number of glycan-degrading enzymes than *Bacteroides* [41]. *Bacteroides* were also significantly correlated with AGS in each of the other gut microbiome studies we examined (Figure 5B-D). For example, *Bacteroides* were abundant in American individuals (mean = 51% relative abundance) where AGS was high (mean = 3.95 Mb), but were less abundant in Swedish individuals (mean = 9% relative abundance) where AGS was lower (mean = 3.1 Mb). Interestingly, AGS and *Bacteroides* abundance differed significantly between nearly all studies we examined (Additional file 11), which may be partially explained by different DNA extraction protocols used by each study. For example, it has been shown that the DNA extraction protocol used by the HMP resulted in greater extraction efficiency for Bacteroidetes relative to the protocol used by the MetaHIT consortium [42].

In the skin and nares communities, we found few strong taxonomic associations with AGS, and our multiple-linear model of bacterial species explained relatively little AGS variation within these body sites (0.13 and 0.03 cross-validation r^2^, respectively). This suggests that the taxa responsible for AGS variation in these communities were not captured by MetaPhlan [43], which was used to estimate the abundance of Bacteria and Archaea. In the oral body sites, we found members of the genera *Mycobacterium* (r^2^ = 0.34), *Bifidobacterium* (r^2^ = 0.31), and *Actinomyces* (r^2^ = 0.33) were all positively correlated with AGS, while members of *Streptococcus* (*Streptococcus infantis* and *Streptococcus mitis*) and of *Haemophilus* (*Haemophilus influenzae* and *Haemophilus parainfluenza*) were negatively correlated with AGS. Lastly, while the posterior fornix was dominated by different *Lactobacillus* spp. (mean relative abundance = 91%), variation in AGS was best explained by members of other genera, including: *Bacteroides*, *Parabacteroides*, *Alistpes*, and *Eubacterium*.

### Average genome size and database coverage are sources of bias for comparative analyses

We hypothesized that the variation in AGS we observed within the human microbiome could significantly bias estimates of gene family abundance from shotgun metagenomes and impact downstream biological analyses. For example, differences in AGS between samples could create the appearance of variation among genes that were present at equal copy number per cell (that is, false positives) and the appearance of stability among genes that varied in copy number per cell (that is, false negatives).

To address this question, we mapped metagenomic reads from 84 HMP stool samples to the KEGG Orthology (KO) Database [38,44] and used these results to compute the relative abundance of protein families in each sample, which is a commonly used metric to estimate gene family abundance from metagenomic data [2,4,33,45,46] (Materials and methods). To validate the accuracy of our functional classifications, we compared our results with results obtained using the HMP Unified Metabolic Analysis Network (HUMAnN) [45]. We found strong concordance between methods, with an average correlation coefficient of 0.91 (all *P*-values = 0) across the samples.

**Figure 6:**
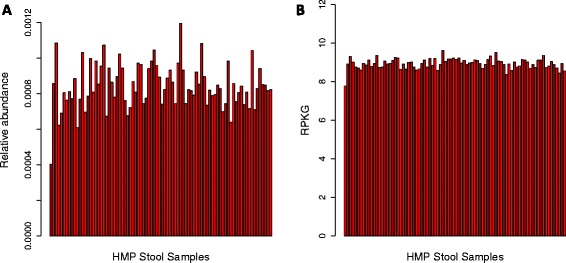
**MicrobeCensus enables accurate quantification of gene family abundance.** Each plot shows the median abundance of essential single-copy genes across 84 stool samples from the HMP. **(A)** Gene family abundance was computed using relative abundance, which is scaled so the abundance of all genes sums to 1.0 for each sample. **(B)** Gene family abundance was computed using RPKG (reads per kilobase per genome equivalent). RPKG leverages estimates of AGS made by MicrobeCensus to normalize gene family abundance values.

Strikingly, we found that the relative abundance of essential single-copy KOs (Additional file 1) varied significantly across stool samples, ranging from a minimum of 4.0e-4 to a maximum of 1.2e-3 (Figure 6A). In other words, genes that were *a priori* known to be present at equal copy number per cell appeared to vary in magnitude by threefold across the study. To identify the source of this bias, we first compared the inter-sample variation of essential KOs to inter-sample variation in AGS. As expected, samples with high AGS had an artificially low abundance of essential single-copy KOs. We found that AGS alone was sufficient to explain approximately 40% of the inter-sample variation of essential KOs (*P* = 7e-11).

However, this still left the majority of the variation unexplained, which prompted us to search for additional sources of bias. We reasoned that differences in database coverage (that is, the fraction of genes in a metagenome that are represented in the reference database) might also result in the appearance of variation among essential single-copy genes. For example, consider two communities: in the first community, 50% of the genes have homologs in a reference database (for example, KEGG Orthology), while in second community, this number is only 25%. Metagenomic sequencing is performed for each community, reads are classified into the reference database, and the relative abundance of gene families is computed. Since relative abundance is the proportion of classified reads mapped to a gene family, we would expect the relative abundance of essential genes to be higher in the community with lower database coverage.

To investigate this possibility, we used the classification rate (that is, fraction of reads classified into the KEGG Orthology Database) as a proxy for database coverage and compared this with inter-sample variation of essential genes. As expected, we found that the classification rate was negatively correlated with the relative abundance of essential genes (ρ = −0.45, *P* = 2e-5). Furthermore, when combined with AGS, these two sources of bias were sufficient to explain 90% of the variation in essential genes across samples (*P* < 2.2e-16). Therefore, differences in both AGS and database coverage should be accounted for when comparing the abundance of genes across samples.

To address these biases, we introduce the measure RPKG (reads per kilobase per genome equivalent) to quantify the abundance of gene families from metagenomic data. RPKG is analogous to the commonly used measure RPKM (reads per kilobase per million sequenced reads) for quantifying transcript abundance in RNA-seq data [47], but instead of dividing by the number of sequenced reads, we divide by the number of genome equivalents, which depends on both library size and AGS (Materials and methods). This measure accounts for gene length, library size, and average genome size. Furthermore, because it is not scaled to sum to 1.0 across gene families, it is not biased by database coverage. If one did normalize RPKG values to sum to 1.0 across gene families, AGS and library size would cancel out in the equation so that the result would be equivalent to the commonly used relative abundance metric (Materials and methods).

We then used RPKG to quantify the abundance of KOs in each of the 84 HMP stool samples. As expected, we found that using RPKG resulted in an 80% decrease in the coefficient variation of essential genes compared with using the relative abundance metric (Figure 6B). Interestingly, we found that it was actually better not to filter duplicate reads when using AGS estimates to quantify RPKG. While we previously found that filtering duplicate reads produced slightly more accurate estimates of AGS, it resulted in 1.6 times more variation of essential genes across samples (Additional file 13). The effect of filtering duplicate reads, and other commonly used quality control procedures, on quantitative metagenomics studies should be investigated in greater detail in the future.

In summary, both AGS and database coverage can be major sources of bias when comparing the relative abundance of genes across samples and should be accounted for in addition to other known biases such as gene length and library size. Our new metric, RPKG, eliminates these unwanted sources of variation and should improve downstream biological analyses. We expect that the biases we observed are not limited to the human microbiome and extend to shotgun metagenomics studies in general.

### Adaptive strategies in the gut microbiome reflect differences in average genome size

Having developed a method to correct for common biases in metagenomic sequencing data, we were interested in exploring variation in the functional composition of the human gut microbiome. Specifically, we sought to better understand the relationship between average genome size and functional ecology in the gut. Previous work has suggested that specialists tend to have smaller genomes than generalists and that organisms adapted to live outside the host have large accessory genomes [8]. However, these observations were made based on a limited number of sequenced gut isolates, and this question has not been directly addressed in the gut microbiome.

To shed light on the relationship between average genome size and functional ecology in the gut, we searched for functions - genes, modules, and pathways from the KEGG Orthology Database - that were strongly correlated with AGS across stool communities within the HMP. Because we used the RPKG metric to quantify gene family abundance, we were confident that the variation we observed was not due to technical biases, and instead reflected true differences in the copy number of genes per cell across microbial communities. We found that AGS tracked with major functional differences across stool samples and was strongly correlated with the first principle component of normalized gene family abundance (that is, RPKG; r^2^ = 0.63, *P* = 3e-19). Furthermore, we found over 1,700 KOs (9% of total), 200 KEGG modules (31% of total), and 150 KEGG pathways (20% of total) whose RPKG was strongly associated with AGS across stool samples (all q < 1e-5) (Additional file 14). Most of these functions tended to be more abundant in communities with larger AGS, as one might expect since genome size and gene content are directly proportional in Bacteria [13]. Together, these findings challenge the notion of functional stability in the human gut microbiome [46,48] and highlight the significant differences in the functional composition of the gut microbiome across individuals.

We found that our normalization procedure was critical to reveal both the magnitude and direction of functional variation among stool samples. Using RPKG enabled us to identify twice the number of positively correlated genes (q < 1e-5, ρ > 0) and likely prevented us from identifying many false positives. For example, when we used relative abundance to quantify gene family abundance, many basic cellular pathways appeared to be differentially abundant, including the ribosome, aminoacyl-tRNA biosynthesis, and RNA polymerase (all q = 0). However, when RPKG was used to quantify gene family abundance, none of these pathways varied significantly across samples (all q > 0.10). In fact, without proper normalization, the major direction of functional variation would have been reversed, with 60% of differentially abundant genes (q < 1e-5) instead being negatively correlated with AGS.

**Figure 7:**
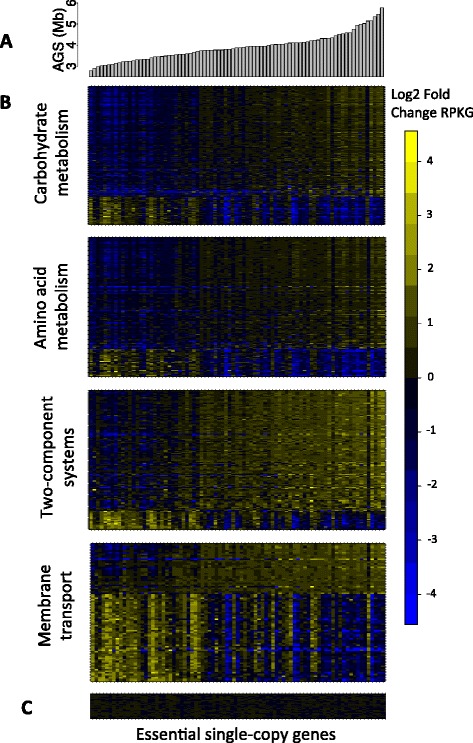
**Average genome size reflects diverse modes of functional adaptation in the gut microbiome. (A)** Barplot of AGS for stool metagenomes. **(B)** Log2 fold change of KOs across stool metagenomes. KOs were grouped according to the BRITE functional hierarchy. Only KOs that were significantly correlated with AGS (*q* < 1e-3) are displayed. **(C)** Log2 fold change of essential single-copy KOs across stool metagenomes.

To better understand this major axis of functional variation in the gut, we sought to identify classes of genes that were commonly associated with AGS. Towards this goal, we performed tests of enrichment using the BRITE functional hierarchy [38], which is an ontology that groups genes that perform related biological functions (Additional file 15). A striking pattern emerged when looking at the top-ranked functional categories from this analysis: genes whose abundances were positively correlated with AGS were enriched in functional categories related to metabolism, biosynthesis, and two-component systems, whereas genes negatively correlated with AGS were enriched in categories related to membrane transport (Figure 7 and Table 1). For example, 27% of genes in two-component systems were positively associated with AGS, compared with only 3% that were negatively associated (q < 1e-5, mean ρ = 0.15); in contrast, 30% of ABC transporter genes were negatively associated with AGS, compared with only 8% that were positively associated (q < 0.01, mean ρ = -0.15). Furthermore, the gene *rpoE*, an ECF-sigma factor involved in regulating expression of polysaccharide utilization loci [38], was strongly correlated with AGS (q = 0, ρ = 0.87) and reached extremely high levels of abundance in the gut.

**Table 1:** **KEGG functions enriched for genes correlated with AGS**

**KEGG function**	**Hierarchy level**	**Direction of correlation**	***q*** **-value**
Metabolism	A	Positive	0
Metabolism of cofactors and vitamins	B	Positive	0
Amino acid metabolism	B	Positive	0
Carbohydrate metabolism	B	Positive	0
Glycan biosynthesis and metabolism	B	Positive	2.7E-05
Two-component system	C	Positive	0
Biosynthesis of amino acids	C	Positive	0
Glycosaminoglycan degradation	C	Positive	1.50E-06
Pentose and glucuronate interconversions	C	Positive	1.07E-05
Lipopolysaccharide biosynthesis	C	Positive	2.21E-05
Histidine metabolism	C	Positive	2.24E-05
Fructose and mannose metabolism	C	Positive	2.69E-05
Carbon metabolism	C	Positive	2.75E-05
Alanine, aspartate and glutamate metabolism	C	Positive	2.89E-05
Biotin metabolism	C	Positive	7.68E-05
Membrane transport	B	Negative	1.0E-05
Drug resistance	B	Negative	6.9E-03
Peptidoglycan biosynthesis	C	Negative	9.5E-10
ABC transporters	C	Negative	2.3E-05
Pyruvate metabolism	C	Negative	7.3E-05

Functions within the BRITE hierarchy were tested for enrichment, using the cumulative hypergeometric test, for genes that were strongly correlated with AGS (Pearson *q* < 1e-5). This was performed separately for genes that were positively correlated and genes that were negatively correlated. Listed are the most significant results from this analysis.

Our results are in agreement with a comparative genomics study which found that *Bacteroides* possess larger genomes and a greater number of glycan-degrading enzymes than *Firmicutes*, which posses smaller genomes but a greater number of ABC transporters [41]. Interestingly, an earlier study [49] found the opposite pattern when looking at the abundance of transporters across 144 complete genomes, suggesting that our result may be specific to the human gut microbiome. Future studies are needed to better understand the relationship between AGS and membrane transport in other environments.

Together, our findings suggest that nutrient acquisition may be accomplished in different ways by different organisms in the gut: organisms with large genomes rely on *de novo* biosynthesis and degradation of complex carbohydrates, whereas organisms with small genomes rely more heavily on transport of simple sugars and amino acids from the environment. Additional regulatory genes, such as sigma factors and two-component systems, are necessary to sense the environment and coordinate expression of the appropriate metabolic and biosynthetic genes.

## Conclusions

MicrobeCensus is a new software tool that rapidly and accurately estimates the AGS of cells in a microbial community from metagenomic data by aligning reads to a set of 30 essential single-copy genes. The accuracy of our method is largely due to extensive optimization of read length-specific alignment parameters, which limit the technical variation of these genes across metagenomes. We performed careful validation of our method, demonstrating that unlike existing methods, MicrobeCensus is able to rapidly and accurately estimate AGS for libraries of varying read length and for communities of varying phylogenetic divergence from sequenced organisms. We found that MicrobeCensus performs well in the presence of Bacteria, Archaea, and Fungi. Therefore, we expect MicrobeCensus to work well in many environments and for many sequencing technologies. However, we found that high levels of viruses resulted in overestimates of AGS, so MicrobeCensus is probably not appropriate for viral metagenomes.

Applying MicrobeCensus to a large number of human microbiome samples, we confirmed that variation in AGS is a major source of bias when comparing the relative abundance of genes across shotgun metagenomes. Additionally, we found that the classification rate (that is, the fraction of reads from a library that are classified into a protein family database) is also a significant source of bias. Together, AGS and the classification rate accounted for 90% of the variation of essential single-copy genes among stool samples from the HMP, which varied from 4.0e-4 to 1.2e-3. To address these issues, we introduced the measure RPKG (reads per kilobase per genome equivalent), to quantify the abundance of gene families from metagenomic data. RPKG accounts for gene length, library size, and average genome size, and is not biased by the coverage of a reference database in a particular environment.

Finally, we found that AGS varies systematically across body sites and individuals, and tracks with important functional and taxonomic differences in the microbiome. For example, in the gut, AGS ranges from 2.5 to 5.8 megabases and is positively correlated with the abundance of *Bacteroides*. After normalizing for AGS and other sources of bias, we found that communities with large AGS were enriched for pathways related to metabolism, biosynthesis, and two-component systems, whereas communities with small AGS were enriched for functions related to membrane transport. These findings highlight how different organisms have developed different adaptive strategies to thrive in the human gut microbiome. These novel observations would have been missed without appropriate normalization.

We expect our approach to be widely applicable. MicrobeCensus is easy to use and can be applied to shotgun metagenomic data from any environment; it is not limited to studies of the human microbiome. We expect the improved accuracy of MicrobeCensus over existing AGS methods to be even greater in environments where fewer genomes have been sequenced (for example, soil, marine). In this study, we focused on shotgun sequenced DNA. In shotgun transcriptomics, similar normalization issues will arise, in conjunction with RNA-specific issues such as the large dynamic range of expression values. Since we expect the essential genes used by MicrobeCensus to be universally expressed, MicrobeCensus could easily be modified to adjust for and investigate the effect of AGS in metatranscriptome studies. To facilitate such extensions, our open-source software is freely available. This work comes at a critical time for the metagenomics field, as more and more studies attempt to quantify and compare relative amounts of many gene families and pathways across samples.

## Materials and methods

### Essential, single-copy gene families

We downloaded a recently published set of 40 phylogenetically diverse, single-copy protein families found in nearly all Bacteria and Archaea [24]. To validate that these genes were in fact universally distributed and single copy, we searched them against the complete proteomes of 250 Bacteria and 79 Archaea (Additional files 1 and 2) using BLAST [50]. An essential gene was deemed present in a genome if its corresponding E-value was below 1E-5 and if the query and target proteins were both covered by at least 70% of their length. We identified a subset of these families (N = 30) with mean copy number close to 1.0, copy number variance close to zero, and universality (fraction of genomes in which gene is present) close to 1.0. Nearly all of these genes (27/30) were components of the ribosome. To address whether these markers could be extended to microbial eukaryotes, we repeated this procedure for 24 complete genomes from Fungi (Additional files 1 and 2). There were an insufficient number of complete genomes from the Integrated Microbial Genomes Expert Review (IMG/ER) database [51] at the time of this writing to evaluate other groups of microbial eukaryotes. While most of these gene families were present in most fungal genomes (21/30 with universality >0.90), they were not as stable as in Bacteria and Archaea. Therefore, a set of universal single-copy genes that covers all three domains of life is needed in future studies.

### Selection of training genomes

We selected 329 diverse genome sequences from Bacteria in Archaea (Additional file 2). These genomes were included in the shotgun sequencing simulations that were used to train our method and were used to validate our 30 essential single-copy gene families. Specifically, we selected 250 complete bacterial genomes from a large 16S gene phylogeny using an algorithm that maximized the total branch length of this sub-tree; additionally we selected 79 complete archaeal genomes that were distinct at the genus level. These genomes ranged in size from 138 kb to 10 Mb and spanned 33 distinct phyla. All reference genomes were downloaded from the IMG/ER database [51].

### Optimizing MicrobeCensus

We developed a modular workflow to train MicrobeCensus given our essential gene families and training genomes. The purpose of this workflow was threefold: 1) to identify the optimal parameters for mapping metagenomic reads to each of the essential gene families at a given read length; 2) to estimate the proportionality constant between AGS and the relative abundance of each essential gene family, given the optimal mapping parameters and read length; 3) to identify weights for each gene family at each read length, such that the weighted average of their individual AGS estimates minimized error in the combined AGS estimate.

The first step in our training workflow was to simulate shotgun sequence libraries in which AGS was known. We used the software tool Grinder [52] to simulate one library from each training genome at each read length, using read lengths ranging from 50 to 500 bp. Specifically, we simulated one 500-bp library for each of the 329 genomes at 200× coverage. For shorter read lengths, we simply trimmed each read from the 3′ end to achieve the desired length. This resulted in 6,580 libraries and totaled over 400 Gb of sequence data.

The next step in our workflow was to identify optimal mapping parameters. We used the tool RAPsearch2 (version 2.10; options: -z 1 -e 1 -t n -p f -b 0) [27] to perform a translated alignment of each simulated library against our database of essential genes. We assigned a read to a gene family if its alignment exceeded a set of mapping parameters that included minimum bit-score, minimum alignment coverage, and maximum percent identity. We tested over 3,000 combinations of these cutoffs. At each read length, we identified the combination of cutoffs that minimized the median unsigned AGS estimation error of each gene across all 329 training libraries.

The next step in our workflow was to identify the proportionality constant between AGS and the relative abundance of an essential gene family at a given read length and using the optimal mapping parameters. First, we used the optimal mapping parameters to assign reads from each training library to the database of essential genes. We used these mapped reads to compute the relative abundance of each of the 30 essential genes in each training library. Relative abundance was computed as the number of mapped reads divided by the library size in base pairs. Because the AGS of each training library was known, the proportionality constant for each gene could be trivially computed for each library. To identify a robust value of *C* for each gene family at each read length, we used the median value of *C* across the 329 training libraries.

Finally, we identified weights for each gene family, such that the weighted average of their individual estimates minimized the median unsigned error in the resulting AGS estimates across the 329 training libraries. To do this we used the R software package optimx [53]. We have provided software necessary to re-run this workflow using user-supplied gene families and training genomes. Our software is freely available at [29]. This may be important as new genomic data become available or for researchers who wish to train MicrobeCensus for microbes from specific environments or using additional gene families; however, retraining will not be necessary for most applications.

### Mock communities and simulated metagenomes for method validation

We generated over 650 metagenomic libraries to test MicrobeCensus under various conditions and to compare performance to existing methods. Each metagenome was composed of reads from 20 bacterial or archaeal genomes that were randomly selected from a pool of over 2,000 complete genome sequences from the IMG/ER database (Additional file 6). To prevent overrepresentation of well-studied organisms (for example, *E. coli*), we selected no more than one genome per named species. Additionally, we excluded the 329 genomes that were used for training MicrobeCensus and genomes of 177 known endosymbionts [51].

Next, we used these genomes to build low, medium, and high complexity communities (Additional file 8). High complexity communities had an equal relative abundance of its 20 members (Shannon entropy = 4.32), whereas low complexity communities were dominated by a single taxon (Shannon entropy = 1.08); medium complexity communities lay between these extremes (Shannon entropy = 2.75).

In addition to these bacterial and archaeal communities, we built communities that contained either microbial eukaryotes or viruses. For eukaryotes, we simulated metagenomes where up to 50% of the genomes came from Fungi, representing up to 94% of total reads. For viruses, we built metagenomes where up to 50% of the reads came from phage genomes. The remaining reads in each metagenome came from an existing medium complexity prokaryotic metagenome. Fungal genomes were randomly selected from the IMG/ER database and phage genomes were randomly selected from NCBI [54] (Additional file 6). Fungal genome sizes ranged from 2.5 to 66.3 Mb with an average of 20.4 Mb, while phage genome sizes ranged from 6.8 to 182.8 kb with an average of 59.5 kb.

The true AGS for each of these communities was determined by taking an abundance weighted average of the community members’ individual genome sizes:

$$ AGS={\displaystyle \sum_{i=1}^n\frac{R_i*{S}_i}{{\displaystyle {\sum}_{i=1}^n{R}_i}},} $$

where *R*_*i*_ and S_*i*_ indicate the relative abundance and genome size of community member *i*. In the case of the viral communities, AGS was determined using only the genome sizes of Bacteria and Archaea since our definition of AGS is based on only cellular organisms.

Next, we used the software package Grinder [52] to simulate metagenomic libraries from each of these communities. The coverage of genome *i* in library *j*, *G*_*ij*,_ was simply a function of the total library coverage *G*_*j*_ multiplied by the organism’s relative abundance: *G*_*ij*_ = *R*_*i*_ × *G*_*j*_. And the number of reads simulated from genome *i* in library *j*, *N*_*ij*_, was simply of function of that genome’s coverage, genome size, and the library’s read length *L*_*j*_: *N*_*ij*_ = (*G*_*ij*_ × *S*_*i*_)/*L*_*j*_. We simulated libraries for a variety of read lengths (50 to 500 bp), sequencing depths (1 to 100× total library coverage), and error rates (0 to 5% uniform error). Sequencing errors were introduced using a 4:1 ratio of substitutions to indels.

Finally, we used MicrobeCensus to estimate AGS for each of these simulated metagenomes using default parameters. These estimates were then compared with the expected value of AGS for the corresponding community. Unless otherwise noted, we measured estimation error as: |*AĜS* - *AGS*|/*AGS*.

### Comparison to existing methods

We compared the estimation accuracy of MicrobeCensus with GAAS (v.0.17) for 20 metagenomes from medium-complexity communities composed of Bacteria and Archaea. These metagenomes contained 100 bp reads, were sequenced at 60× total coverage, and contained no sequencing error. For GAAS, we first searched the reads against the database of microbial genomes included with the software (NCBI RefSeq release 56) using BLAST (v.2.2.26) with the following options: blastall -m 8 -p blastn. We supplied the BLAST output to GAAS using the following command: gaas -f query.fna -d reference.fna -m blast.m8 -a taxon.map -v nucleic -e 1e-03 -sm 0 -j 1 -gp 0 -gs 0 -gt 0. In parallel, we ran MicrobeCensus using default parameters on the same 20 simulated metagenomes.

To simulate the presence of novel taxa, we held back reference sequences belonging to organisms from the same taxonomic group as organisms in the metagenome. We used each method to estimate AGS using only the remaining reference sequences. For MicrobeCensus, the reference sequences included the 30 gene families, whereas the reference sequences for GAAS were complete microbial genomes. We performed this procedure and evaluated performance at each taxonomic level: species, genus, family, order, class, and phylum. For example, at the genus level, this procedure would discard alignments between *E. coli* shotgun sequences and all reference sequences from *Escherichia*; at the phylum level, this procedure would discard alignments between *E. coli* shotgun sequences and all reference sequences from Proteobacteria.

Separately, we evaluated whether the method described in Raes *et al*. was able to accurately estimate average genome size for modern libraries composed of short reads. To this end, we used the same 20 mock communities that we previously used when benchmarking GAAS. Here, however, we simulated libraries with read lengths ranging from 50 to 500 bp, where each library was simulated at ≥20× coverage. These libraries were simulated without sequencing error. Because no software was released, we had to manually implement the method described by Raes *et al*. To this end, we downloaded version 3 of eggNOG database [55] and identified all protein sequences that corresponded to the 35 single-copy orthologous groups (OGs) used by Raes *et al*. Next, we created a BLAST database of these sequences using the tool formatdb and searched each simulated library against the 35 OGs with BLASTX (v.2.2.26). We only assigned a shotgun sequence to one of the 35 OGs if the alignment score exceeded 60 bits but the percent identity was less than or equal to 50%. We allowed a read to hit multiple OGs, conditional that the alignments overlapped by no more than 50% of the shorter sequence. Finally, we computed marker density (x) as the number of hits per megabase of input data, and used the Raes formula to estimate average genome size: Average genome size = a + (b × L^-c^)/x, where a = 21.2, b = 4230, c = 0.733, and L = library read length (Raes *et al*. refer to this measure as effective genome size). We ran MicrobeCensus on the same data and computed estimation error for both methods as previously specified.

### Estimation accuracy using real data

To evaluate the ability of MicrobeCensus to estimate average genome size from real shotgun libraries, we downloaded short-read Illumina datasets from 42 completed microbial isolate genome projects using SRAdb [56] (Additional file 7). After converting the SRA files to FASTQ format, we used FASTQC [57] to identify adapter contamination in each dataset, and used cutadapt [58] (-e 0.1 --discard -O 5) to remove any contaminated sequences. Additionally, we removed exact duplicates using the first end of each paired-end read, and filtered reads with an average quality score of less than 5. Next, we pooled these data to create 10 mock metagenomes. Each metagenome was composed of 5 million 70-bp reads from 10 randomly selected genome projects (that is, 500,000 reads from each project). The true AGS of each metagenome was computed as previously described. We used MicrobeCensus to estimate AGS for each of these 10 mock metagenomes. Finally, we evaluated the effect of various quality control procedures on AGS estimation accuracy. Specifically, we evaluated adaptor filtering, duplicate filtering, filtering by mean read quality, filtering by minimum read quality, and filtering reads with ambiguous base calls (that is, Ns; Additional file 9).

### Speed benchmarking

We benchmarked the speed of MicrobeCensus, GAAS, and the Raes method on a simulated 150-bp shotgun sequence library, which contained between 1,000 and 1 million reads. GAAS was run with default options, except -v nucleic -sm 0, to increase its speed. MicrobeCensus was run with default options, except -t to specify the number of threads to use. The Raes method was run as previously described. All tests were performed on a server with 16 Intel Xeon X5560 2.80 GHz CPUs and 200 Gb of RAM running Ubuntu 10.04.4 LTS.

### Minimum number of reads for precise AGS estimates

To determine the minimum number of reads needed to precisely estimate AGS, we ran MicrobeCensus using different numbers of randomly selected reads from several real datasets. Specifically, we chose Human Microbiome Project samples from eight different body sites (SRS011084, SRS017849, SRS023847, SRS019029, SRS042984, SRS043663, SRS019127, SRS023468). For each sample, we selected up to 20 million unique, single-end reads trimmed to 70 bp and searched these reads against the database of essential genes using RAPsearch2. Next, we used bootstrapping to repeatedly estimate AGS with MicrobeCensus using random subsets of these 20 million reads. We evaluated sample sizes of 10,000 to 20 million reads. For each sample size we performed 100 bootstrap iterations. This enabled us to estimate the amount of dispersion (variance/mean) observed for each sample at a given number of sampled reads (Additional file 4).

### Minimum number of genes for accurate AGS estimates

To determine whether the 30 genes we selected were sufficient for low-error AGS estimates, we ran MicrobeCensus on 20 simulated prokaryotic metagenomes (100-bp error-free reads; 60× coverage), using between 1 and 30 essential genes (Additional file 5). For each number of genes used, we took a weighted average across the estimates produced by each gene. We used up to 30 random combinations of genes at each level.

### Human microbiome sequence data

We downloaded 738 metagenomic samples from the Human Microbiome Project on 22 September 2013 from the HMP Data Analysis and Coordination Center (HMPDACC) [59]. Additionally, we downloaded metagenomic data for Chinese individuals [5] from the NCBI Sequence Read Archive (SRA045646, SRA050230) on 22 December 2013; for Spanish and Danish individuals from the MetaHIT project [1] from the Beijing Genomics Institute [60] on 13 January 2014; and for Swedish individuals [6] from the NCBI Sequence Read Archive [ERP002469] on 7 February 2014.

### Estimating AGS for human microbiome samples

We used MicrobeCensus to estimate AGS for human microbiome samples using up to 5 million single-end reads trimmed to 70 bp (Additional file 10). Additionally, we used options to remove duplicates (-d), filter reads with mean quality less than 5 (-m 5), and filter reads containing more than 5% unknown base calls (-u 5). In all downstream analyses, we only considered samples for which there were at least 300,000 reads after our quality control procedures. When using RPKM to measure gene family abundance, we estimated AGS without filtering duplicate reads (Additional file 13). Other options used were the same as before.

### Abundance of KEGG Orthology groups

We selected 84 HMP stool samples for functional analysis. Each sample was from a different subject, and when there were multiple samples per subject, we chose the sample from the subject’s first clinic visit. We excluded one outlier sample (SRS016585) from our analyses that we discovered was composed of only *E. coli* (96.4%). Next, we downsampled the first 10 million reads of at least 75-bp from each FASTQ file, using only the first end of each paired-end read. Next, we used the tool Prodigal [61] (v.2.60) to identify the likely protein coding sequence from each read (command: prodigal -i input.fa -a output -p meta -q). These protein sequences were searched against the KEGG Orthology Database (March 2013) using RAPsearch2 [27] (v.2.22) with the command: rapsearch -q input.faa -d kegg.db -o output.m8 -i 30 -v 1 -b 0 -t a. After filtering alignments with bit scores less than 30, we assigned each read to the KO according to its top scoring hit.

Based on these top scoring hits, we then computed the abundance of each KO in each sample. First, we computed abundances using our AGS-normalized measure, called RPKG. The RPKG of a KO in a metagenome was computed by: 1) counting the number of reads mapped to the KO; 2) dividing (1) by the length of the KO in kilobase pairs; and 3) dividing the result of (2) by the number of sequenced genome equivalents:

$$ RPKG=\frac{Mapped\; reads/ Gene\; Length\;(Kb)}{Genome\; equivalents}, $$

where:

$$ Genome\; equivalents=\frac{Library\; size\;(bp)}{AGS\;(bp)}, $$

and library size is the total number of sequenced base pairs. To compare our measure, we also computed the relative abundance of each KO in each sample. The only difference between these measures is that relative abundance is scaled so that the sum across KOs for a sample is equal to 1.0:

$$ Relative\; Abundance=\frac{Mapped\; Reads/ Gene\  Length\ \left(\mathrm{kb}\right)}{{\displaystyle \sum Mapped\; Reads/ Gene\  Length\ \left(\mathrm{kb}\right)}}. $$

For higher levels in the ontology, we summed the abundance of all KOs that mapped to a particular module or pathway.

To validate our mapping approach, we compared the relative abundances of KOs obtained from our pipeline to that obtained using the software HUMAnN [45]. HUMAnN relative abundances were downloaded from the HMP Data Analysis and Coordination Center [59]. For each sample, we computed a linear regression of the relative abundance of KOs between the two methods.

### Abundance of taxonomic groups

For Bacteria and Archaea, we downloaded MetaPhlAn [43] taxonomic annotations for Human Microbiome Project samples on 21 December 2013 from the HMP Data Analysis and Coordination Center. For other stool microbiomes, we ran MetaPhlAn v.1.7.7 using the options: --bowtie2db bowtie2db/mpa --input_type = multifastq -t rel_ab.

### Statistical analyses

To identify genes that tracked with average genome size, we performed Pearson correlations between the abundance of KOs - using both relative abundance and RPKG - and AGS. We used the false discovery rate (FDR) procedure [62] to correct for multiple testing. Next, we performed cumulative hypergeometric tests to identify functional categories within the BRITE hierarchy that were enriched for genes that were highly correlated with AGS. We used an FDR adjusted *P*-value cutoff of 1e-5 to determine whether a gene was correlated or not. We performed this procedure separately for genes that were positively and negatively correlated with AGS. We used the FDR procedure to correct hypergeometric *P*-values.

## Additional files

Additional file 1:
**A table listing the 30 essential single-copy genes used to estimate average genome size along with their copy number distributions across Bacteria, Archaea, and Fungi.**
Additional file 2:
**A table listing the 329 phylogenetically diverse bacterial and archaeal genomes used to validate the 30 essential genes and used in shotgun sequencing simulations to train MicrobeCensus.**
Additional file 3:
**Shows the effect of parameter tuning on the ability to accurately estimate AGS.**
Additional file 4:
**A figure that shows the effect of sequencing depth on dispersion of AGS estimates from real metagenomes.**
Additional file 5:
**Shows AGS estimation error as a function of the number of marker genes used.**
Additional file 6:
**A list of the bacterial, archaeal, eukaryotic, and viral genomes used in metagenomic simulations to test MicrobeCensus and existing methods.**
Additional file 7:
**A table listing the completed genome projects from NCBI that were used to build mock Illumina metagenomes.** AGS estimates for these projects are also included. Additional file 8:
**A figure that shows the effect of community complexity on AGS estimation error.**
Additional file 9:
**A figure that illustrates the effect of various sequence quality filters on AGS estimation accuracy.**
Additional file 10:
**A table listing AGS estimates for human microbiome data.**
Additional file 11:
**Lists** ***P*** **-values from comparisons of AGS between body sites, oral subsites, and gut microbiome projects.**
Additional file 12:
**Lists associations between AGS and bacterial and archaeal taxa within the HMP dataset.**
Additional file 13:
**Shows the effect of duplicate filtering on AGS estimates from HMP stool samples and the downstream effect on RPKG.**
Additional file 14:
**A table listing correlation coefficients for functions associated with AGS across stool samples from the HMP.**
Additional file 15:
**A table listing BRITE functional categories that were enriched for KOs significantly correlated (either positively or negatively) with AGS across HMP stool samples.**

## Abbreviations

AGS: average genome size
bp: base pair
FDR: false discovery rate
HMP: Human Microbiome Project
IMG/ER: Integrated Microbial Genomes Expert Review
KEGG: Kyoto Encyclopedia of Genes and Genomes
KO: KEGG Orthology group
Mb: megabase
OG: orthologous group
RPKG: reads per kilobase per genome equivalent
RPKM: reads per kilobase per million sequenced reads
SRA: Sequence Read Archive
