# Supplementary material for: Average genome size estimation improves comparative metagenomics and sheds light on the functional ecology of the human microbiome
Source: Genome Biol. 2015 Mar 25;16(1):51. doi: 10.1186/s13059-015-0611-7 (PMC4389708; doi:10.1186/s13059-015-0611-7)
Supplement: Additional file 3: — Shows the effect of parameter tuning on the ability to accurately estimate AGS. [file 13059_2015_611_MOESM3_ESM.pdf]

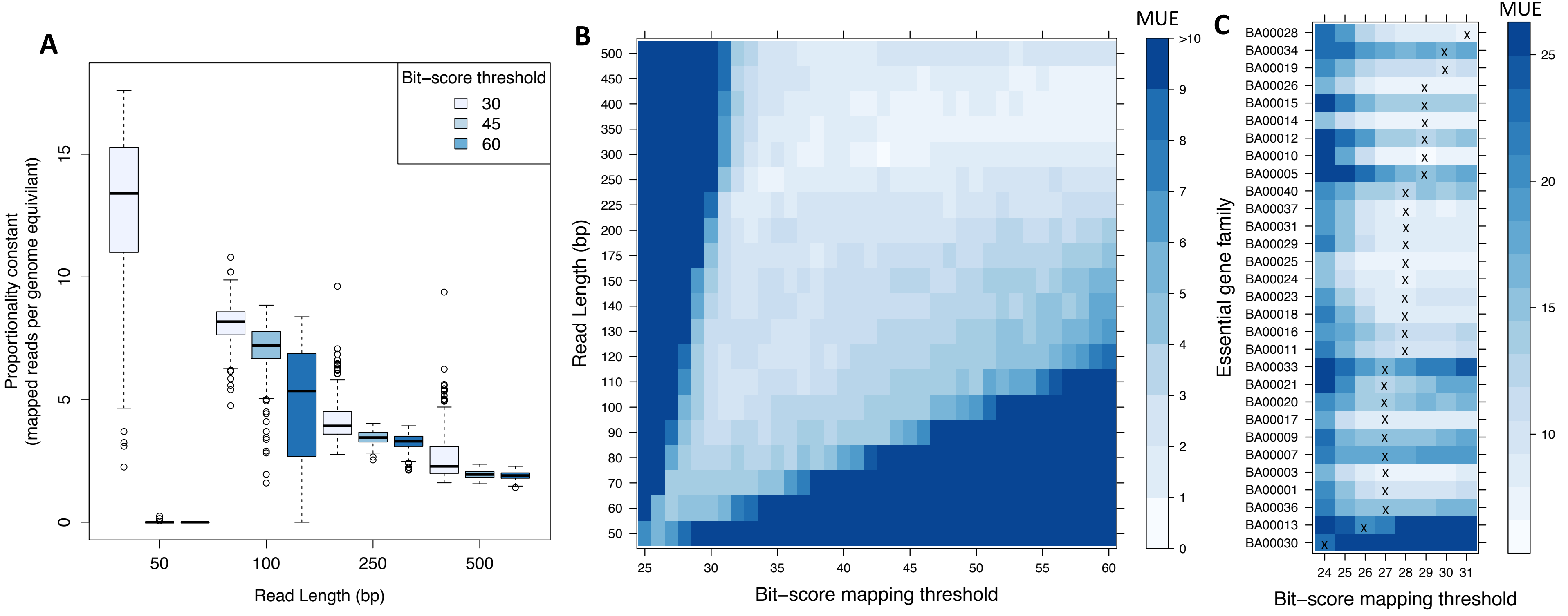

Accurate estimation of AGS depends on read length and gene family specific mapping parameters. a) Distribution of proportionality constants for gene family BA00010 across 329 training libraries. Proportionality constants depend upon read length and mapping parameters. **b)** Median unsigned error (MUE) for gene family BA00010 across 329 training libraries. AGS estimation error is minimized when using read-length specific mapping parameters. **c)** MUE for all 30 gene families across 329 70-bp training libraries. Gene families vary in their usefulness for estimating AGS. Error is minimized when using gene-family specific mapping parameters.
