# Supplementary material for: Average genome size estimation improves comparative metagenomics and sheds light on the functional ecology of the human microbiome
Source: Genome Biol. 2015 Mar 25;16(1):51. doi: 10.1186/s13059-015-0611-7 (PMC4389708; doi:10.1186/s13059-015-0611-7)
Supplement: Additional file 4: — A figure that shows the effect of sequencing depth on dispersion of AGS estimates from real metagenomes. [file 13059_2015_611_MOESM4_ESM.pdf]

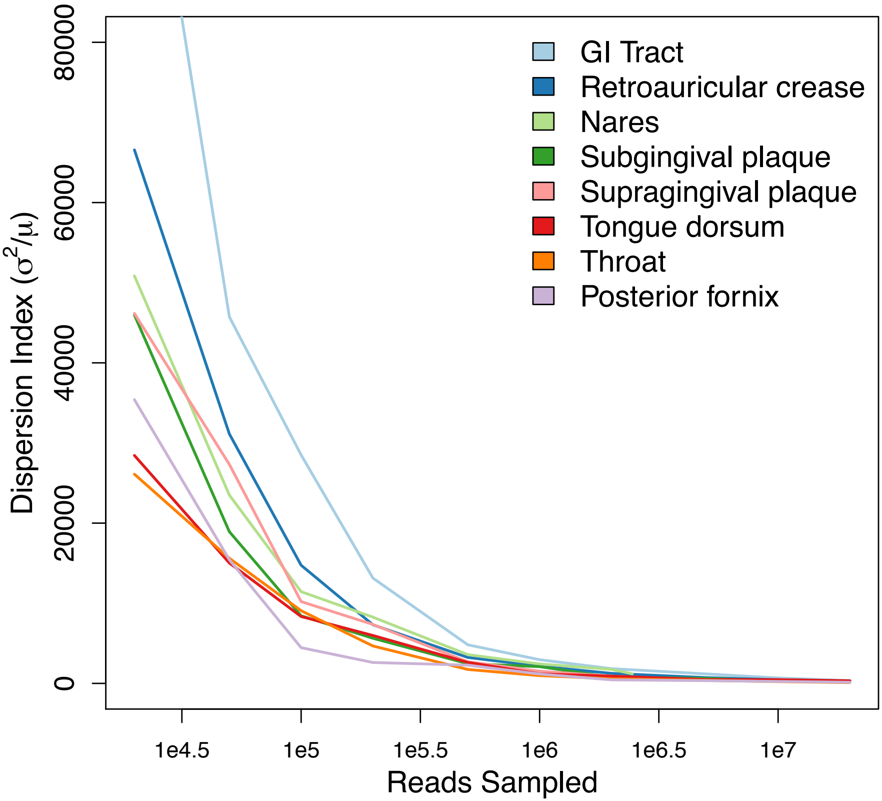

500,000 reads are sufficient for low variance estimates of AGS for most metagenomes. MicrobeCensus was run using between 10,000 and 20 million randomly sampled reads from 8 HMP samples (one per body-site). At each sampling depth, we performed 100 bootstraps in order to estimate the amount of dispersion (i.e. variability in AGS estimates).
