# Supplementary material for: Average genome size estimation improves comparative metagenomics and sheds light on the functional ecology of the human microbiome
Source: Genome Biol. 2015 Mar 25;16(1):51. doi: 10.1186/s13059-015-0611-7 (PMC4389708; doi:10.1186/s13059-015-0611-7)
Supplement: Additional file 8: — A figure that shows the effect of community complexity on AGS estimation error. [file 13059_2015_611_MOESM8_ESM.pdf]

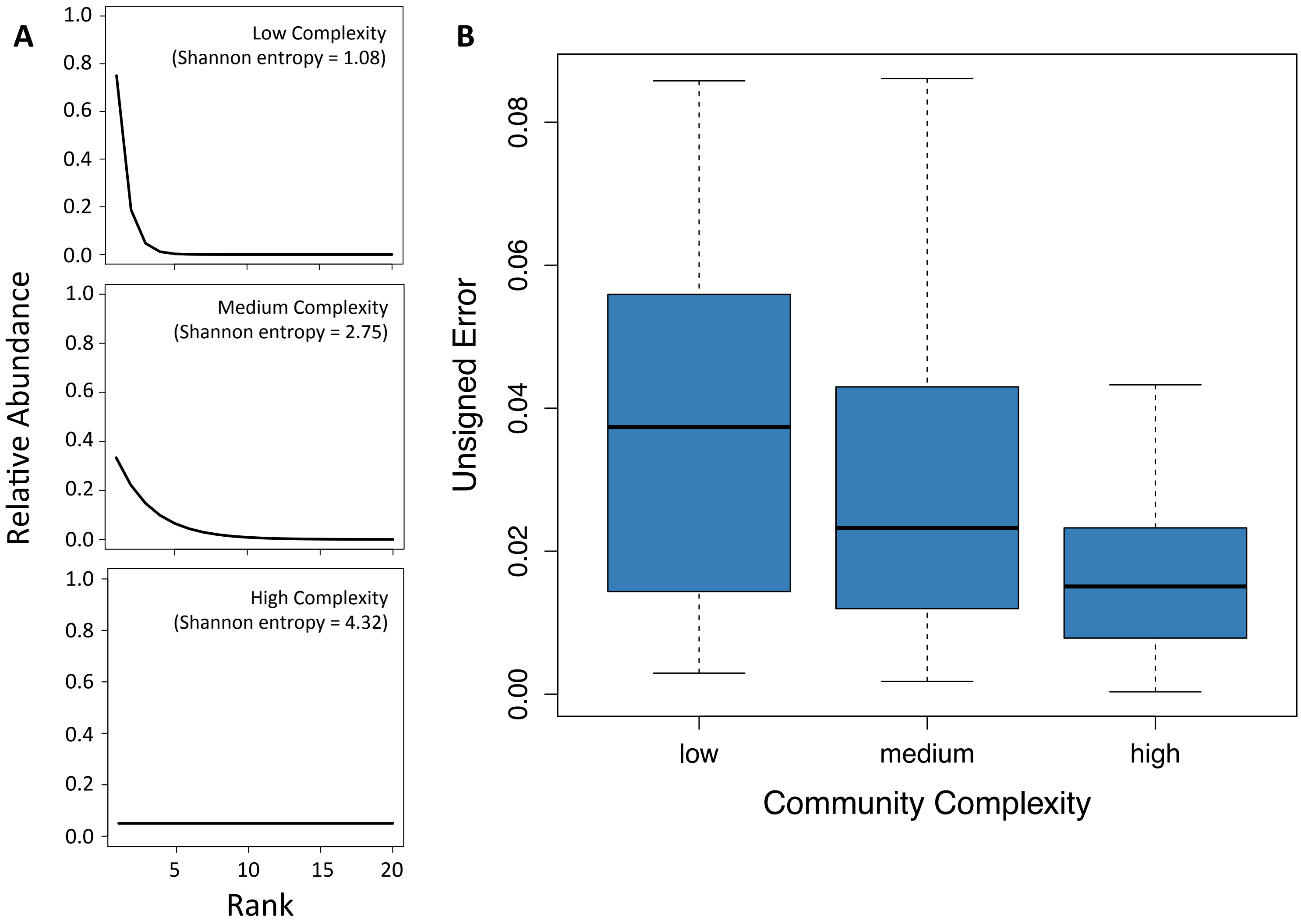

The effect of community complex on AGS estimation error. a) Low, medium, and high complexity mock microbial community were constructed. 20 communities of each type were constructed. Each community contained 20 randomly selected prokaryotic genomes. 100-bp metagenomes were simulated from each community. **b)** AGS was estimated for each of the metagenomes described in (a). Shown are distributions of unsigned estimation error for each community type. Unsigned estimation error is lower for higher complexity communities, likely because *signed* errors cancel out for each of the individual taxa present in the community.
