# Supplementary material for: Average genome size estimation improves comparative metagenomics and sheds light on the functional ecology of the human microbiome
Source: Genome Biol. 2015 Mar 25;16(1):51. doi: 10.1186/s13059-015-0611-7 (PMC4389708; doi:10.1186/s13059-015-0611-7)
Supplement: Additional file 9: — A figure that illustrates the effect of various sequence quality filters on AGS estimation accuracy. [file 13059_2015_611_MOESM9_ESM.pdf]

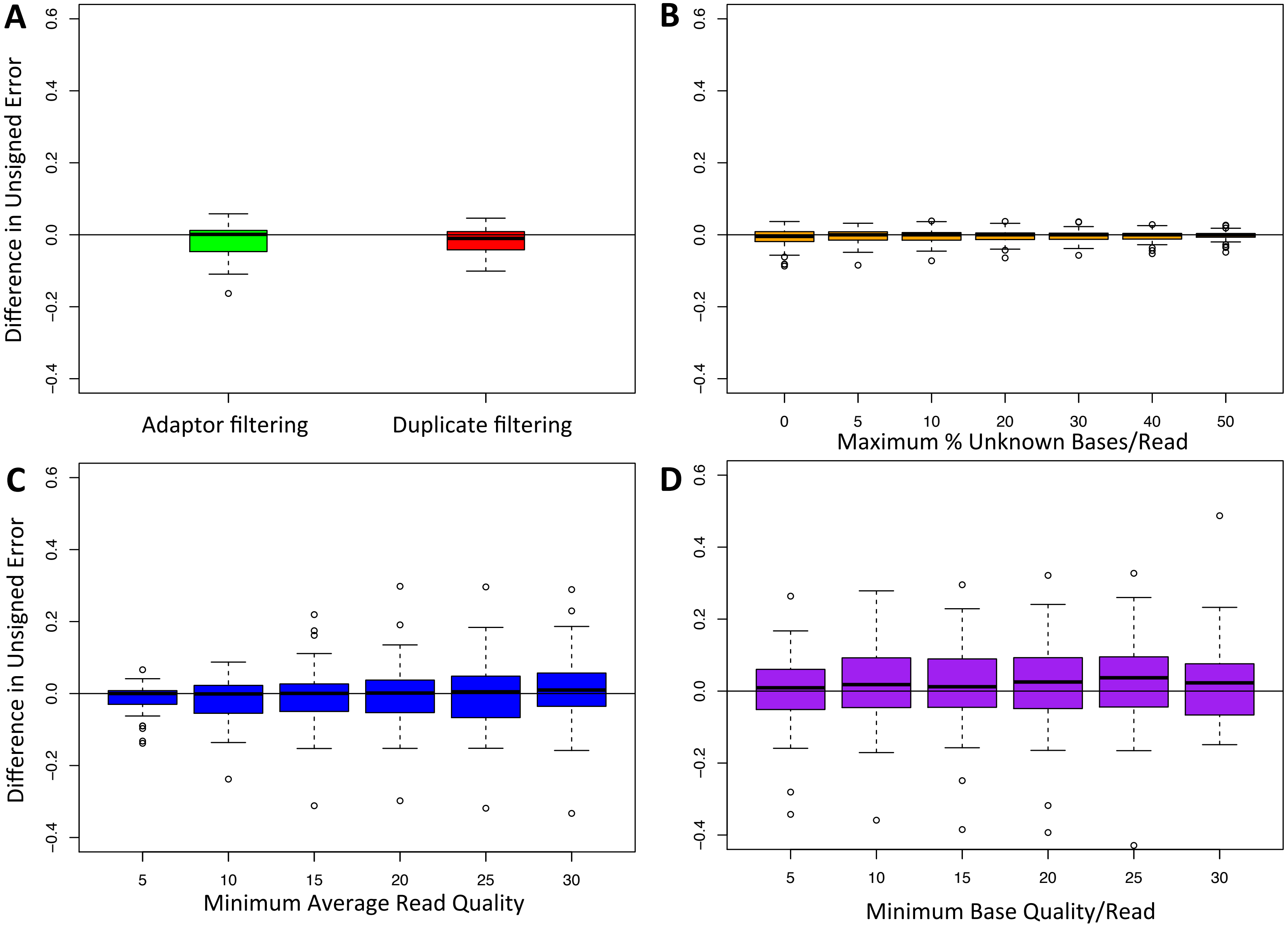

Effect of quality control filters on accurate estimation of AGS. We used MicrobeCensus to estimate genome size for 42 short-read Illumina genome projects using various quality control filters. Plotted are the differences in error with and without using the quality filter. **(a-b)** There is a consistent, but small reduction in error when removing adaptor contamination, filtering duplicate reads, and filtering reads that contain unknown base calls. **(c)** There is a small reduction in error when filtering the lowest quality reads (minimum average quality < 5). **(d)** When filtering reads by their minimum quality score, there is an overall increase in prediction error.
