# Supplementary figures and images for: Average genome size estimation improves comparative metagenomics and sheds light on the functional ecology of the human microbiome
Source: Genome Biol. 2015 Mar 25;16(1):51. doi: 10.1186/s13059-015-0611-7 (PMC4389708; doi:10.1186/s13059-015-0611-7)

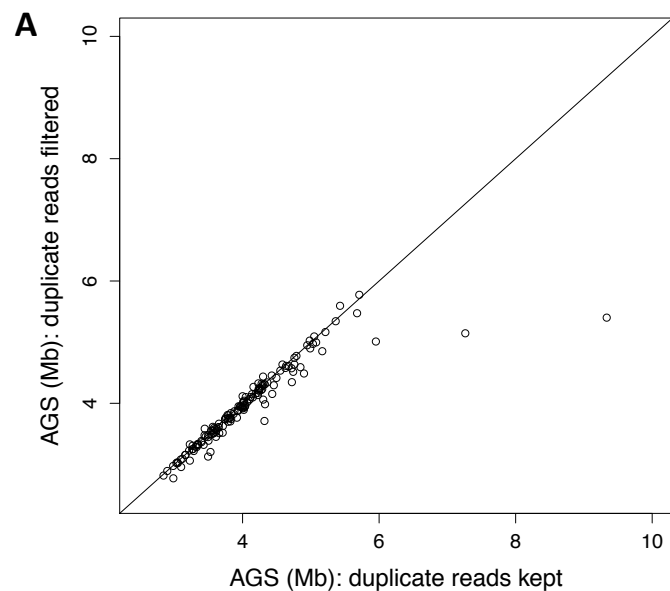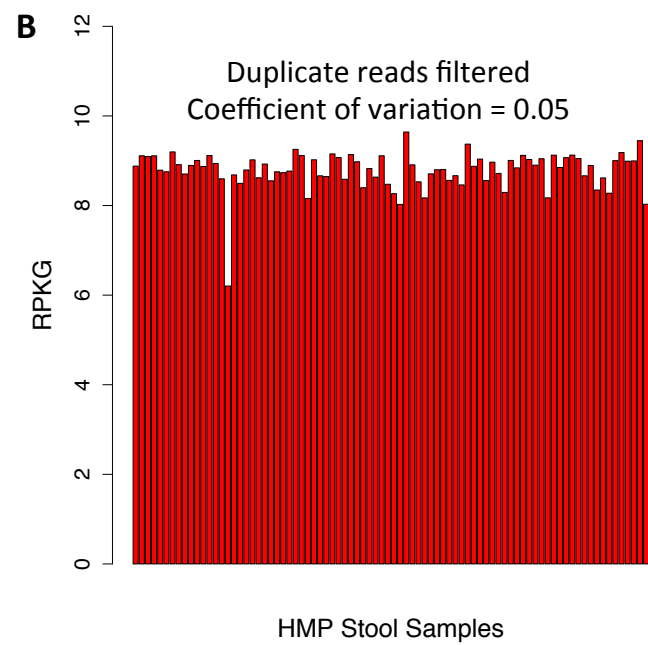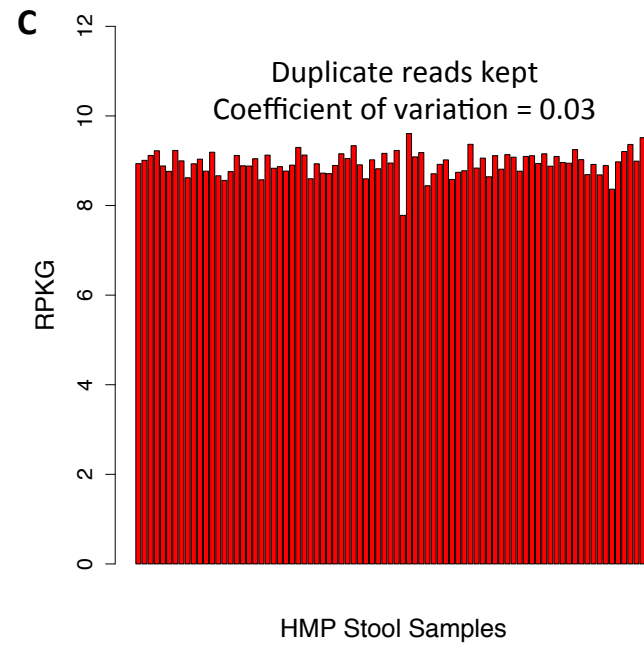

Supplement: Additional file 13: — Shows the effect of duplicate filtering on AGS estimates from HMP stool samples and the downstream effect on RPKG. [file 13059_2015_611_MOESM13_ESM.pdf]
